# Oenothein B in Eucalyptus Leaf Extract Suppresses Fructose Absorption in Caco-2 Cells

**DOI:** 10.3390/molecules27010122

**Published:** 2021-12-26

**Authors:** Keiichiro Sugimoto, Midori Amako, Hiroaki Takeuchi, Kazuya Nakagawa, Morio Yoshimura, Yoshiaki Amakura, Tomoyuki Fujita, Shigeo Takenaka, Hiroshi Inui

**Affiliations:** 1 Research and Development Center, Nagaoka Co., Ltd., Ibaraki, Osaka 567-0005, Japan; htakeuchi@npc-nagaoka.co.jp (H.T.); knakagawa@npc-nagaoka.co.jp (K.N.); 2 Center for Research and Development of Bioresources, Osaka Prefecture University, Sakai, Osaka 599-8570, Japan; amakom@bioinfo.osakafu-u.ac.jp; 3 Department of Pharmacognosy, College of Pharmaceutical Sciences, Matsuyama University, Ehime, Matsuyama 790-8578, Japan; myoshimu@g.matsuyama-u.ac.jp (M.Y.); amakura@g.matsuyama-u.ac.jp (Y.A.); 4 Department of Agriculture, Graduate School of Science and Technology, Shinshu University, Nagano 399-4598, Japan; tfujita@shinshu-u.ac.jp; 5 Department of Nutrition, College of Health and Human Sciences, Osaka Prefecture University, Habikino, Osaka 583-8555, Japan; takenaka@rehab.osakafu-u.ac.jp (S.T.); inui@biochem.osakafu-u.ac.jp (H.I.); 6 Department of Health and Nutrition, Otemae University, Osaka 540-0008, Japan

**Keywords:** eucalyptus leaf extract, fructose absorption, oenothein B, polyphenol, hydrolyzable tannins, ellagitannin, glucose transporter 5, Caco-2 cell, polyvinylpolypyrrolidone

## Abstract

Inhibition of fructose absorption may suppress adiposity and adiposity-related diseases caused by fructose ingestion. Eucalyptus leaf extract (ELE) inhibits intestinal fructose absorption (but not glucose absorption); however, its active compound has not yet been identified. Therefore, we evaluated the inhibitory activity of ELE obtained from *Eucalyptus globulus* using an intestinal fructose permeation assay with the human intestinal epithelial cell line Caco-2. The luminal sides of a cell monolayer model cultured on membrane filters were exposed to fructose with or without the ELE. Cellular fructose permeation was evaluated by measuring the fructose concentration in the medium on the basolateral side. ELE inhibited 65% of fructose absorption at a final concentration of 1 mg/mL. Oenothein B isolated from the ELE strongly inhibited fructose absorption; the inhibition rate was 63% at a final concentration of 5 μg/mL. Oenothein B did not affect glucose absorption. In contrast, the other major constituents (i.e., gallic acid and ellagic acid) showed little fructose-inhibitory activity. To our knowledge, this is the first report that oenothein B in ELE strongly inhibits fructose absorption in vitro. ELE containing oenothein B can prevent and ameliorate obesity and other diseases caused by dietary fructose consumption.

## 1. Introduction

Fructose consumption has increased in the past five decades because of the use of high-fructose corn syrups as sweeteners in beverages and processed foods [1,2]. Sucrose is also ingested in large amounts, as it is traditionally used in many foods and beverages [3]. Excess fructose intake induces adiposity [1,2,4,5], fructation (a kind of glycation caused by amino-carbonyl reactions with fructose) [5,6], and insulin resistance [2,5,7,8], which can lead to the development of nonalcoholic fatty liver disease (NAFLD) [9,10], diabetes mellitus [1,7], and hypertension [1,11]. Malik et al. have reported that consumption of beverages sweetened with sugar and/or artificial sweeteners is positively related to mortality [12]. The World Health Organization has suggested that the consumption of such sugars should be reduced to less than 10% of the total energy intake but insists that it is desirable to reduce it to within 5% (roughly 25 g (six teaspoons) per day) in a published guideline for sugar intake [13].

Fructose is more lipogenic than glucose in the liver because its metabolic pathway does not include the step catalyzed by 6-phosphofructokinase, the main rate-controlling enzyme in glycolysis; therefore, large amounts of ingested fructose can be rapidly metabolized without metabolic regulation [14]. Fructose serves as the carbon source for both the glycerol and acyl portions of triacylglycerol molecules, leading to de novo lipogenesis [2,5].

Fructose is passively incorporated from the luminal side of the intestine across membranes by glucose transporter 5 (GLUT5), one of the facilitative GLUT family members [15]. GLUT5 is the sole fructose-specific transporter and does not transport glucose or galactose; transapical transport of fructose in the intestine is mediated primarily via GLUT5 [11,15]. Furthermore, GLUT5 plays an initial role in the development of hypertension induced by fructose consumption [11]. A clinical trial on isocaloric fructose restriction in diets demonstrated that children with obesity showed a consistent decrease in de novo lipogenesis in the liver within a short period. Subsequently, their hepatic and visceral fat levels were improved [16]. In a similar study in obese children, glucose and lipid metabolism were improved [17]. In a trial, diets of overweight adults with fatty liver were supplemented with glucose or fructose daily. After 6 weeks, a significant decrease in the intrahepatic lipid content was observed in the glucose group compared to the fructose group [18]. A restriction of fructose intake in the diet for 24 weeks reduced waist circumference and fasting blood glucose concentration in overweight and obese adults [19]. Therefore, inhibiting GLUT5 could be an important strategy for preventive medicine or for therapeutic intervention for the foregoing diseases caused by excessive fructose ingestion.

The evergreen tree *Eucalyptus globulus* Labill (Myrtaceae), native to Tasmania, Australia, is globally distributed. Eucalyptus leaves are used to prepare herbal tea in Europe [20] and are traditionally used as a natural remedy for diabetes mellitus in South America and Africa [21]. The extract of the leaves (eucalyptus leaf extract; ELE) has recently been used as a functional food or in cosmetics because of its beneficial effects, such as antioxidative [22,23], anticaries [24], and anti-skin-aging properties [25]. The safety of ELE was reported by the Japanese Ministry of Health, Labor and Welfare based on the results of several mutagenesis assays, mouse bone marrow micronucleus assays, and a 90-day repeated dose toxicity study [26]. We previously reported the safety assessment of oral ELE administration for 4 weeks in adult men [27]. Furthermore, we reported that ELE inhibits fructose absorption in the intestine and suppresses the accretions of visceral fat and hepatic triacylglycerols induced by excessive fructose ingestion in rats [28]. This inhibitory effect on intestinal fructose absorption has also been observed in human subjects [29]. However, the inhibitory constituent of fructose absorption in ELE has not yet been identified. Therefore, we aimed to identify the active constituents of ELE by performing an in vitro intestinal fructose absorption assay using the human intestinal epithelial cell line Caco-2.

## 2. Results and Discussion

### 2.1. Inhibitory Activities of ELE on Fructose Absorption

Dietary fructose is incorporated into the intestinal tract through GLUT5 at the apical side of the cells and transported to the portal vein via GLUT2 at the basolateral side [15,30]. Caco-2 cell line spontaneously differentiates into enterocytes, which possess various characteristics of the small intestine, such as brush border membrane enzymes and nutrient transporters, including GLUT5 [31]. The cell line has been used in various fructose transport studies [32]. We examined the effects of ELE and its constituents on fructose permeation across an intestinal epithelial cell monolayer model from the luminal side to the basolateral side using Caco-2 cells to identify the active constituent of the extract. Before evaluating the inhibitory effect of ELE on fructose absorption using Caco-2 cells, we confirmed that GLUT5 regulates fructose absorption in this model; we verified that cytochalasin B [33], a GLUT2 inhibitor, does not affect fructose absorption at a final concentration of 100 μmol/L (the inhibitory rate on glucose absorption was 31%).

ELE strongly inhibited fructose absorption at a final concentration of 1 mg/mL in Caco-2 cells (Table 1). At this dose, ELE-treated cells did not show different morphological features, as confirmed by transepithelial/endothelial electrical resistance (TEER) and microscopic analyses; before and after fructose permeation, the TEER values of the blank and ELE (1 mg/mL) were retained by 91% and 81%, respectively. Many studies have reported various polyphenols derived from edible plants with inhibitory activity against fructose absorption [32,34,35,36,37,38,39,40,41,42,43]. We examined the effects of the ELE with reduced polyphenolic substances. Polyvinylpolypyrrolidone (PVPP) has high affinity for polyphenols, and, therefore, the removal of PVPP using centrifugation, following the treatment, removes polyphenols from the extract [44]. PVPP treatment considerably reduced the total polyphenol content and PVPP-treated ELE (yield, 26%) showed weak inhibition on intestinal fructose absorption (Table 1), suggesting that polyphenols were the main active constituents of the ELE.

### 2.2. Identification of the Fructose Absorption Inhibitor in ELE

**Figure 1:**
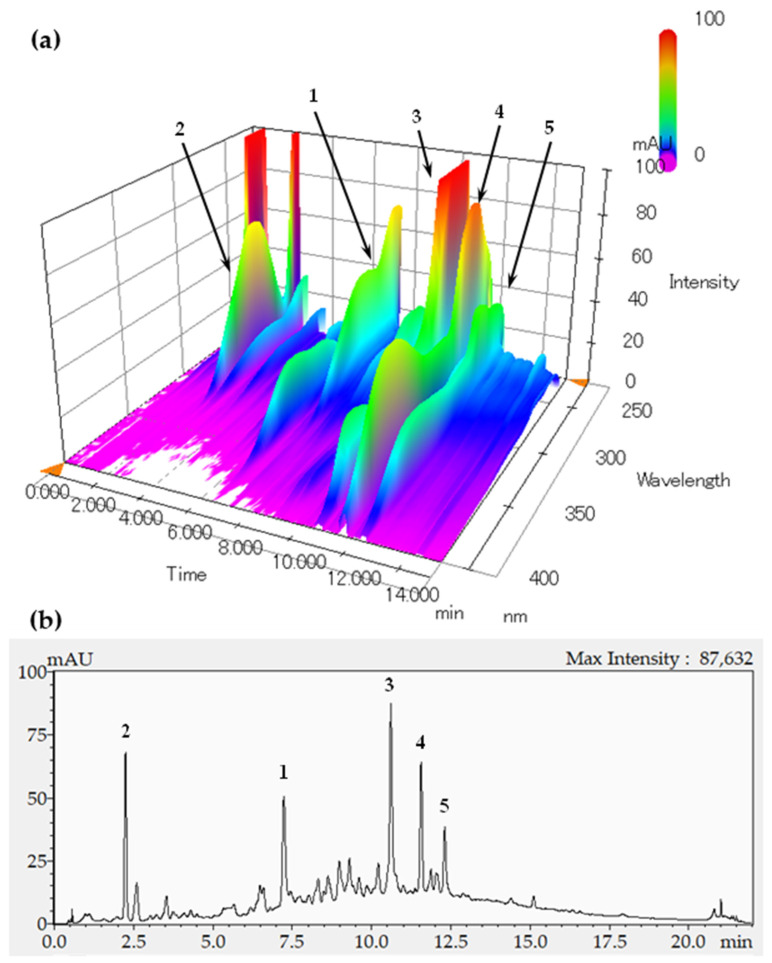
HPLC profiles of ELE recorded for UV absorption from 220 to 400 nm (**a**) and at 270 nm (**b**). The retention times of compounds **1**–**5** were 7.24, 2.25, 10.52, 11.56, and 12.31 min, respectively.

**Figure 2:**
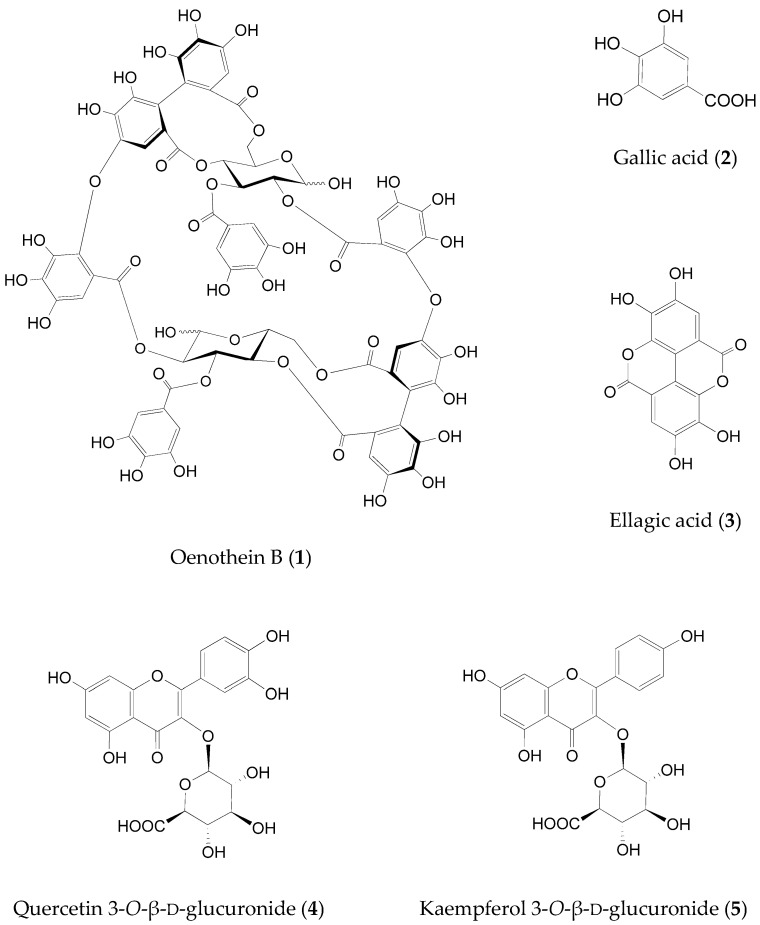
Chemical structures of the major constituents of the ELE.

The profiles of the ELE constituents were analyzed using HPLC with a diode-array detector (DAD). Figure 1a shows the three-dimensional chromatogram between 220 and 400 nm. Among the detected compounds, oenothein B (**1**), gallic acid (**2**), ellagic acid (**3**), quercetin 3-*O*-β-d-glucuronide (**4**), and kaempferol 3-*O*-β-d-glucuronide (**5**) were identified as the major constituents of ELE with a similar profile as that presented in a previous report [23]. Figure 2 shows the chemical structures of the compounds **1**–**5**. We evaluated the inhibitory activity of these constituents on fructose absorption (Table 2). Among them, compound **1** strongly inhibited fructose absorption. We also evaluated the effect of **1** on glucose absorption, because ELE administration barely affected the postprandial increase in blood glucose levels in vivo [28,29]. This constituent did not affect glucose absorption (data not shown).

Compound **1** is a hydrolyzable tannin characterized by the presence of a glucopyranose core that is esterified by polyphenolic acids, such as a hexahydroxydiphenoyl (HHDP) unit and compound **2**. Tannins, including compound **1**, have diverse biological effects, such as antioxidant, anti-inflammatory, antitumor, enzyme inhibitory, and antimicrobial effects [45]. To our knowledge, compound 1 is the first to be identified as an inhibitor of fructose absorption from hydrolyzable tannins.

Compounds **2** and **3**, which are hydrolysates of hydrolyzable tannins, barely inhibited fructose absorption. Compounds **4** and **5** also showed a similar trend. Satsu et al. have reported that compound **2** and quercetin (aglycon of compound **4**) did not inhibit fructose incorporation into Caco-2 cells, whereas kaempferol (aglycon of compound **5**) showed a weak inhibition [41]. We also examined the inhibitory effect of quercetin on fructose permeation and found little inhibition (less than 30%) at a dose of 50 μg/mL.

Gonçalves et al. stated that modulating intestinal fructose absorption via GLUT5 and/or GLUT2 might provide a novel pharmacologic strategy for diseases caused by excessive fructose consumption [30]. Recent studies have shown that ingestion of fructose beyond the fructose clearance capacity of the intestine leads to leakage of incorporated fructose into the portal vein, thus forcing the liver to metabolize the excess fructose to produce fat. When an appropriate amount of fructose is taken up by individuals, it is converted to other substances by metabolic enzymes, such as ketohexokinase, in the intestinal tract, and de novo lipogenesis in the liver is mitigated [46]. Restriction of fructose intake improved lipid metabolism and obesity in certain dietary intervention trials [16,17,18,19]. Therefore, limiting fructose intake even slightly might be beneficial for people who consume excessive amounts of fructose. Many studies have reported that polyphenol intake reduces the risk of diseases, such as obesity and type 2 diabetes mellitus. Loureiro et al. suggest that inhibition of intestinal absorption of glucose and fructose is a potent mechanism that could help manage these diseases [40]. Although the inhibitory effects of compound **1** on intestinal fructose absorption need to be confirmed in in vivo experiments, ELE containing **1** is expected to have preventive and inhibitory effects on fructose-derived obesity and related diseases.

We previously reported that compound **1** is a major constituent and a significant chemical marker of the *E. globulus* leaf; it is a rare natural product and can be detected easily without interference from other peaks on the ELE chromatogram [23]. We considered that compound **1** may be the main constituent of the ELE possessing inhibitory activity for fructose absorption. 

**Figure 3:**
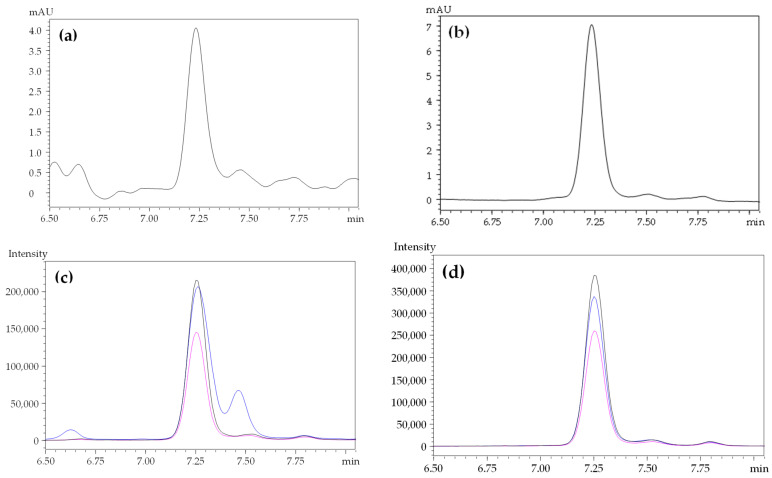
Comparisons of the UV chromatograms and MS/MS profiles of ELE and the purified oenothein B (**1**). (**a**,**c**), ELE; (**b**,**d**), purified compound **1**. (**a**,**b**) show HPLC chromatograms recorded at 270 nm. (**c**,**d**) show MS/MS profiles in the negative ion mode at *m*/*z* 765.00 (black), 300.85 (blue), and 275.00 (pink).

Next, we attempted to develop a quantitative analysis method for compound **1**. The peak for compound **1** appeared almost independently in the chromatogram of the ELE recorded at 270 nm (Figure 1b), although a small contamination peak was observed around the retention time of 7.5 min. We isolated compound **1** as an analytical standard from ELE using preparative methods described previously [23]. The peak for compound **1** was identified using HPLC coupled to electrospray ionization mass spectrometry (ESI-MS). The peak for compound **1** appeared around 7.24 min in the chromatograms of the ELE (Figure 3a) and the isolated compound **1** (Figure 3b). The MS/MS fragmentations of the peaks that appeared around 7.24 min also showed almost the same patterns upon the comparison of the following ions: [M-2H]^2−^ at *m*/*z* 783.00 as a precursor ion and product ions at *m*/*z* 765.00, 300.85, and 275.00 [47,48] between the ELE (Figure 3c) and isolated compound **1** (Figure 3d).

Baert et al. [47] reported the UV calibration curve of **1** with linearity for concentrations ranging from 0.3 to 150 μg/mL (R^2^ = 0.9984) and a limit of quantitation (LOQ) of 0.88 μg/mL. However, solutions of the isolated substance showed large variabilities in the low-concentration group (<10 μg/mL). Although we speculate that **1** in high-purity and low-concentration solutions is nonspecifically adsorbed onto the surfaces of the experimental tubes in a manner similar to the adsorption of unstable peptides [49], further research is necessary in this regard. In the future, we may establish a validated method for the quantitative analysis of ELE.

## 3. Materials and Methods

### 3.1. Materials

The leaves of *E. globulus*, used as the test material, were obtained from K. Kobayashi & Co. (Kobe, Japan). The extract of 100 g of dried leaves was obtained using 1 kg of ethanol–water (1:2, *v*/*v*) under reflux for 2 h. The obtained extract was filtered, evaporated to dryness in vacuo, and used as ELE throughout the experiments. The yield was approximately 20% of the leaves. HPLC- and LC/MS-grade solvents were purchased from Nacalai Tesque, Inc. (Kyoto, Japan). Compounds (Figure 2; oenothein B (**1**), quercetin 3-*O*-β-d-glucuronide (**4**), and kaempferol 3-*O*-β-d-glucuronide (**5**)) used as test samples in the sugar absorption assays were isolated from ELE [23]; cytochalasin B and gallic acid (**2**) were purchased from Fujifilm Wako Pure Chemical Co. (Osaka, Japan) and ellagic acid (**3**) from Sigma-Aldrich Japan (Tokyo, Japan). Other reagents mentioned elsewhere in the text with the manufacturer’s name not specified were purchased from Nacalai Tesque, Inc. (Kyoto, Japan) or Fujifilm Wako Pure Chemical Co. (Osaka, Japan).

### 3.2. Cells

The human intestinal epithelial cell line Caco-2 was obtained from Dainippon Sumitomo Pharma (Osaka, Japan). The cells were cultured according to a previous method (described in Section 27.5.4 of that report [50]). BD Falcon cell culture inserts and 6-well cluster plates (BD Biosciences, Franklin Lakes, NJ, USA; Catalogue No. 353090) were used for cultivation of Caco-2 cells and the fructose absorption assay. In brief, subcultured cells were allowed to reach confluence in Dulbecco’s modified Eagle’s medium (DMEM; Sigma-Aldrich Japan, Tokyo, Japan; Catalogue No. D5796) containing 10% fetal calf serum (Biowest, Nuaillé, France) and 1% nonessential amino acids (Sigma-Aldrich, Tokyo, Japan; Catalogue No. M7145), and seeded on the inside of the insert at a density of 2 × 10^5^ cells/insert. The DMEM was changed both apically (inside the insert) and basolaterally (the plate) every 3–4 days. Cells were allowed to differentiate for 3 weeks.

### 3.3. Measurement of Fructose and Glucose Absorption Inhibitory Activity

After confirming the purity to be >95% by reversed-phase HPLC, the isolated compounds **1**, **4**, and **5** were used for subsequent experiments. Each sample solution was prepared by dissolution of test sample in 10% dimethyl sulfoxide.

The fructose absorption assay was conducted according to a previous method (described in Section 27.5.4 [50]). Briefly, both the inside and outside of the inserts were washed with 1 mL of phosphate-buffered saline without magnesium and calcium (pH 7.4). Dulbecco’s phosphate-buffered saline (1 mL; pH 7.4; Gibco, Langley, OK, USA; catalog no. 21300-025), which is glucose-free, was used as a medium for the absorption experiment, and was added to the plate and the inside of the insert. The cells were incubated for 30 min at 37 °C in a humidified incubator containing 5% CO_2_. Monolayer formation by Caco-2 cells was confirmed by measuring TEER of the medium between the plate and in the inside of the insert using the Millicell-ERS Voltohmmeter (Millipore, Bedford, MA, USA). Next, 10 μL of sample solution was added to the medium on the inside of the insert, followed by incubation for 5 min at 37 °C in a humidified incubator containing 5% CO_2_. Subsequently, 10 μL of 100 mM fructose solution (final concentration, 1 mM) was added to the medium on the inside of the insert, and the cells were incubated for an additional 3 h at 37 °C at the same conditions. The TEER was measured again, and the medium in the plate was collected and stored at −20°C in a sterile tube. The fructose concentration in the medium was analyzed using a previously described fructose-dehydrogenase-based method [28,50]. To examine the inhibitory effect of the sample on glucose absorption, we added 100 mM glucose, instead of fructose, solution to the medium in the inside of the insert, after 10 μL of the sample solution was added and preincubated for 5 min at 37 °C in a humidified incubator containing 5% CO_2_. The plates were incubated for 3 h under the same conditions as those of the fructose absorption assay. The glucose concentration (final concentration, 1 mM) in the medium was analyzed using the Glucose CII Test Wako kit (Fujifilm Wako Pure Chemical Co., Osaka, Japan).

### 3.4. Treatment of ELE with PVPP

An ELE (1 g) aliquot was mixed with 10 g PVPP (Polyclar VT; ISP Japan, Tokyo, Japan) in 30% ethanol (*v*/*v*). This mixture was stirred at room temperature (25 °C) for 1 h. Subsequently, the mixture was centrifuged at 1000× *g* for 15 min at 25 °C and filtered. A PVPP-treated ELE was obtained by concentration in vacuo and lyophilization of the filtrate (yield, 26%). 

### 3.5. Measurement of Total Polyphenol Content

The total polyphenol content of ELE and PVPP-treated ELE was measured in terms of gallic acid equivalents according to a previously described method [51] with modifications. Test samples and gallic acid were dissolved in purified water and sonicated for 5 min. Anhydrous sodium carbonate was used for preparing 20% Na_2_CO_3_ solution (*w*/*v*). Sample solution (500 μL) was mixed with 500 μL of Phenol Reagent Solution (acid degree, 1.8 N; Nacalai Tesque, Inc., Kyoto, Japan), and 1.5 mL of 20% Na_2_CO_3_ solution was added immediately. After vortexing, each solution was allowed to stand at room temperature (25 °C) for 20 min, after which the absorbance was respectively measured at 720 nm.

### 3.6. HPLC-DAD-ESI-MS Analysis of ELE

The samples were dissolved in a water/methanol (50:50; *v*/*v*) solution and filtered through a TORAST Disc GLCTD-HPTFE1322 hydrophilic PTFE membrane filter (pore size, 0.22 μm; Shimadzu GLC Ltd., Kyoto, Japan). The filtrate was added to a glass vial (TORAST-H Glass Vial; Shimadzu GLC Ltd., Kyoto, Japan) and set on a vial tray in the autosampler at 4 °C. ELE analysis was conducted using Shimadzu Nexera X2 (Shimadzu Co., Kyoto, Japan) equipped with SPD-M30A (Shimadzu Co., Kyoto, Japan) as DAD according to previously reported methods [23,52] with slight modifications. The conditions of LC using a Cosmosil 5C18-PAQ packed column (Nacalai Tesque, Inc., Kyoto, Japan; 5 μm, 150 mm × 2.0 mm i.d.) were as follows: column temperature, 40 °C; flow rate, 0.7 mL/min; and injection volume, 3 μL. UV spectra were recorded over a range of 220–350 nm and chromatograms were acquired at 270 nm. The time programs of the mobile phase were conducted with a linear gradient in reversed-phase mode using a water/formic acid (1000:1; *v*/*v*) solution as solvent A and acetonitrile as solvent B. The gradient program was 0–16% B in A in 9 min, 16–45% B in A in 11 min, 45–95% B in A in 1 min, 95% B in A for 2 min, and 95–100% B in A in 1 min.

The identification of compound **1** was conducted according to previously reported methods [47,48]. The mass spectroscopy equipment LCMS-8050 (Shimadzu Co., Kyoto, Japan) was connected to Shimadzu Nexera X2 following DAD in series. The LC eluate was introduced directly into the ESI interface without splitting. ESI-MS was operated in the negative ion mode using Ar as the collision gas at 350 kPa with the following settings: probe voltage, −2.5 kV; nebulizing gas (N_2_) flow, 3.00 L/min; drying gas (N_2_) flow rate, 10 μL/min; curved desolvation line voltage, default values; curved desolvation line temperature, 200 °C; heating gas (N_2_) flow rate, 10 L/min; block heater temperature, 450 °C; interface temperature, 400 °C; and analysis mode, multiple reaction monitoring. The identification of **1** (*m*/*z* 783.00) representing [M-2H]^2−^ was confirmed by comparing the observed molecular ions and their retention times. The product ions *m*/*z* 765.00 (collision energy, 23 V), 300.85 (collision energy, 43 V), and 275.00 (collision energy, 39 V) obtained from *m*/*z* 783.00 as a precursor ion were also analyzed for the identification.

## 4. Conclusions

Taken together, the findings of this study showed that oenothein B (**1**), a major constituent and a significant marker characteristic to *E. globulus* leaves, is an active constituent of the ELE and inhibits fructose absorption in intestinal cells, as observed during an in vitro intestinal permeability model assay. To the best of our knowledge, this is the first study to report that oenothein B in ELE strongly inhibits intestinal fructose absorption. ELE can be manufactured stably and inexpensively, owing to the ease of availability of the *E. globulus* leaves as the ELE source since this species is distributed worldwide. ELE containing oenothein B is expected to prevent and ameliorate obesity and diseases such as NAFLD, diabetes mellitus, and hypertension that are caused by the dietary consumption of fructose. However, the amount of oenothein B in ELE could not be assessed. In the future, we wish to validate a method for the quantitative analysis of ELE.

## Figures and Tables

**Table 1:** Total polyphenol content and inhibitory effects of ELE on fructose absorption.

Sample	Total Polyphenols (%)	Dose (mg/mL)	Inhibition ^1^ (%)
ELE	30	1	65
PVPP-treated ELE	5	0.26	<20

^1^ Each value represents the mean of two experiments.

**Table 2:** Inhibitory effect of constituents of eucalyptus leaf extract on fructose absorption.

Sample	Dose (μg/mL)	Inhibition ^1^ (%)
Oenothein B (**1**)	5	63
Gallic acid (**2**)	50	<20
Ellagic acid (**3**)	50	<30
Quercetin 3-*O*-β-d-glucuronide (**4**)	50	<30
Kaempferol 3-*O*-β-d-glucuronide (**5**)	50	<30

^1^ Each value represents the mean of two experiments.

## Sample Availability

Samples of the compounds are not available from the authors.
